# Lithium: Old Drug, New tricks?

**DOI:** 10.1007/s11920-025-01646-0

**Published:** 2025-10-15

**Authors:** Rebecca Strawbridge, Christiana Boules, Richard Morriss, Allan H. Young

**Affiliations:** 1 Department of Psychological Medicine, Institute of Psychiatry, Psychology & Neuroscience, King’s College London, London, UK; 2 South London & Maudsley NHS Trust, London, UK; 3 Faculty of Medicine & Health Sciences, University of Nottingham, Nottingham, UK; 4 Dept of Brain Sciences, Head of Division of Psychiatry, Imperial College London, London, UK

**Keywords:** Lithium, Adverse effects, Safety, Effectiveness, Dose, Titration, Monitoring

## Abstract

**Purpose of Review:**

Lithium is considered to have substantial benefits and harms, which constitutes a complex clinical challenge. This article aims to cover five putative strategies to maximise the benefits of lithium therapy while concurrently minimising its harms.

**Recent Findings:**

The strategies range in terms of their evidence base. Some are supported by long-standing evidence, namely the management of some common and/or severe adverse effects. For others, we are seeing emerging research focus, particularly in the use of lithium in ‘sub-therapeutic’ doses. In considering gradual titration and variable dosing, as well as innovation to enhance the monitoring of lithium, there is limited evidence which is reviewed.

**Summary:**

We conclude that optimising lithium therapy is not straightforward and clearly individual patient characteristics must be thoroughly prioritised. However, a general rule for optimising can be to maintain lithium at its lowest therapeutic level for an individual patient, with close monitoring of levels and adverse effects as well as careful titration where clinically indicated.

## Introduction

Lithium has long been regarded as the gold standard in the treatment of bipolar disorder, offering significant efficacy in both acute mood episodes and long-term relapse prevention [1]. Its therapeutic benefits extend beyond bipolar disorder to include unipolar depression, suicidality prevention, and emerging evidence for its potential in neuroprotection and cognitive health [2]. This broad therapeutic spectrum highlights lithium’s importance in psychiatric care, making it an essential component of clinical guidelines worldwide [3].

However, the use of lithium is not without challenges. At high therapeutic doses, and particularly with long-term use, lithium is associated with significant risks, including renal impairment, thyroid dysfunction, and neurological side effects [2, 4]. These adverse effects necessitate close monitoring of renal and thyroid function, which can be burdensome for both patients and healthcare systems [5]. Additionally, lithium’s narrow therapeutic window raises concerns about toxicity, which can result in life-threatening complications if blood levels are not carefully managed [2].

Beyond the physiological concerns, treatment adherence is a critical determinant of lithium’s effectiveness. Despite its benefits in stabilising mood and reducing suicidality, adherence rates to lithium therapy remain suboptimal, and are most often (up to 62%) due to perceived side effects [6]. Barriers to optimal monitoring and adherence together not only compromise individual outcomes but also exacerbate the cost-benefit considerations of long-term lithium more broadly [2].

In light of these challenges, there is growing interest in optimising lithium therapy, not only in exploring potential additional indications but also through dose adjustment, more substantive management of its associated risks and innovative monitoring strategies. Emerging evidence suggests that lower doses of lithium can, in some circumstances, retain efficacy while reducing the risk of adverse effects, presenting a promising avenue for maximising therapeutic benefits [2].

This article considers five potential approaches to address the known drawbacks of lithium treatment, which are not mutually exclusive. Some are more strongly supported by evidence than others, and we attempt to make this clear. If evidence across these can be advanced further, it can help to ensure that this cornerstone of psychiatric treatment remains accessible and effective for all who may benefit from it.

Both for the selection of the five approaches included, and the contents of each approach, the following methodological approaches were taken: Key extant works in the field were examined, as were both their reference and citation lists; these reviews were supplemented with separate database searches of PubMed using relevant terms and consensus as to the material included was reached between the authors.

Before progressing further, we emphasise that there is no one-size-fits-all approach across these topics. In some people, pre-lithium assessment and subsequent monitoring should be more frequent/in-depth e.g., in the context of high lithium levels, lifestyle changes, medical concerns or concomitant treatments. Indeed, special considerations and/or adjustments should be made for certain groups of people. This includes but is not limited to those with particular concerns around weight gain and/or polydipsia, those who might become pregnant, those with needle/blood phobia, those taking medication to be used in caution with lithium, those with adherence challenges and suicidality.

## Adjunct Therapies to Manage Side Effects

It is firstly relevant (see subsequent sections) that there is a dose association between lithium and its adverse effects. For most, frequency/severity increases notably above serum lithium 0.7/0.8mmol/L [6]. For some (e.g., diarrhoea, tremor, weight gain) complaints are most frequent in early treatment stages and do not tend to worsen with longer term treatment [6]. Others (see below) can decline with longer durations of lithium use.

In a large examination of reasons for lithium discontinuation, the most common was adverse effects and the most common adverse effects noted as reasons for discontinuing were diarrhoea (13%), tremor (11%), polyuria and/or polydipsia (9%), increases in creatinine (9%) and weight gain (7%) [6]. Of these, management strategies for common side effects align with those recommended in general (not specifically for lithium) e.g., taking medication alongside a meal for gastrointestinal effects [3], recommendations for managing weight gain [3]. This section does not comprehensively cover these or many other adverse effects including in cardiovascular, CNS, dermatological and endocrinological domains, though assessment and management guidance is available and recommended for consultation [3].

Before focusing more on three particular areas of adverse effect (tremor, renal, thyroid), we briefly mention that patients should be informed about the importance of water and salt intake: due to lithium’s effects on their metabolism, changes in intake can lead to medical complications in those at risk e.g., in people with a history of normal pressure hydrocephalus, this may increase the risks of raised intracranial blood pressure or a complication leading to lithium toxicity. While this risk primarily applies to those who are prone to circulatory difficulties (e.g., heart failure), it can also affect others exposed to dehydration or sunstroke [3].

### Tremor

Tremor is a common side effect that can occur even in the presence of low-level lithium. It is suggested that tremor is most common in the early stages of lithium treatment and becomes less prevalent in those treated for longer [7]. Tremor varies widely in severity (as well as type) [7]. To emphasise its clinical importance, tremor can be an indicator for lithium toxicity, though it can also be triggered by concomitant agents or comorbidities. Assessment, including for parkinsonism, is important. In addition to addressing other factors (e.g., thyroid, other medication use, anxiety disorders, substance use, essential tremor etc.), beta-blockers, primidone or other anticonvulsants can be beneficial if required [8]. In fact, lamotrigine, valproate, carbamazepine and quetiapine are examples of these which clearly have mood stabilising effects themselves. A general recommendation (see more below) is to adjust doses so that mood stabilising medications are at a minimal therapeutic level can be helpful and this may be especially important for antipsychotics where a therapeutic level in bipolar disorders is lower than for pure psychotic illness.

### Renal

Best known and most severe of lithium’s adverse effects relates to renal health. Renal function appears negatively associated with both dose and duration of lithium treatment and is less readily manageable than those above [7]. In instances of creatinine increase or more relevantly, decreasing estimated glomerular filtration rate (eGFR), guidelines are available though management options are limited. Guidelines include advice in terms of seeking nephrology input and in the absence of other strategies, the subject of down-titrating or discontinuing lithium has been widely discussed [3, 4]. Polyuria is common and may be mild or more severe in cases of nephrogenic diabetes insipidus (NDI). It has long been advised to treat polyuria/NDI with amiloride [9], however the evidence remains extremely scant despite clearly positive findings [10] and understood mechanisms of action [3].

### Thyroid

Common, potentially severe, and eminently treatable adverse effects relate to the thyroid. Hypothyroidism is most common, with levothyroxine (T4) as the mainstay treatment. Parathyroidism is also a risk following increases in calcium with lithium treatment [11] and the most common intervention options are calcimimetics e.g., cinacalcet [12] (see also below). Lithium does not carry known risks for hyperthyroidism or other associated disorders [3]. Hypothyroidism and/or elevated thyroid stimulating hormone in lithium treated patients may be associated with BD severity or rapid cycling BD, and in these patients treatment with thyroxine may also improve affective outcomes [13]. It may be worth mentioning that hypothyroidism in lithium-treated patients may not reflect genuine thyroid dysfunction, and instead be related to iodine uptake [14]. As such, it can be useful to examine other symptoms suggestive of hypothyroidism e.g., cold intolerance or slow pulse. Finally, when considering intervening with thyroid test abnormalities, it is recommended to first conduct repeated tests, since single measures of thyroid function can be unrepresentative of an ongoing issue where intervention is indicated. Increased parathyroid hormone is associated with a greater illness burden in people with bipolar disorder, but is not always associated with increased calcium [11]. Since both can exacerbate, potentially independently, health conditions (renal, other medical, and psychiatric [15]), it is important to examine both and consider other potential causes. This may require endocrinology referrals.

Box 1 summarises one real-world clinical case demonstrating the importance of thorough thyroid examination.

**Table:** 

Case 1:
*0.6-0.6.8mmol/l levels*,* well controlled and stable over extended period. Sudden onset rapid cycling*,* substantially elevated TSH plus low/normal free T3 and T4 and without other symptoms suggestive of hypothyroidism. 50 mg levothyroxine stabilised TSH levels and rapid cycling condition. After extended period of euthymia and high functioning*,* 6 years later*,* sudden worsening of bipolar illness (hypomanic and depressive episode plus frequent cycling); emergency visit with drowsiness and disorientation. After initial assessment for lithium toxicity (without elevated lithium level)*,* diagnosis of dehydration. After correction*,* hypercalcemia identified and then elevated parathyroid hormone. Endocrinologist diagnosed primary hyperparathyroidism. Full recovery and remained on lithium after surgery.*

The key summary messages for this section are that firstly, adverse effects are not necessarily a sufficing reason to avoid lithium, and that secondly it is important to address adverse effects particularly as they can have concerning consequences for physical and/or mental health.

## Use of Low-level Lithium

Our understanding of lithium’s narrow therapeutic index originates from the seminal 1954 study by Schou et al. [16], where doses were increased after the point of manic resolution until toxicity (diarrhoea, nausea, tremor) was observed. Doses were then lowered, observing reversed toxicity and maintained clinical benefit. Since then, studies have largely supported the use of lithium at 0.6-0.6.8mmol/L (usually for treating depression or bipolar maintenance), and 0.8–1.8.0mmol/L (usually for treating mania) and studies have overwhelmingly employed these ‘therapeutic’ levels. Regardless, adverse effects at these levels are not negligible and lower levels appear associated with far reduced adverse effects [2]. We briefly summarise below clinical evidence focused on *low level* (< 0.6mmol/L) lithium. We acknowledge that this is not comprehensive, and note that we do not include here studies using low dose lithium without reporting serum levels. When discussing levels, note that these are lithium levels 12 h after the most recent dose [17].

### Depression

This evidence is observational and taken largely from bipolar populations [18] although there is small-scale support for low-level lithium adjunctively in unipolar depression [19] and people prescribed lithium for depression are at risk of developing bipolar. Despite a lack of controlled trial evidence, efficacy studies generally aim for a minimum lithium concentration of 0.5–0.6 mmol/L [20].

### Suicidality

One clinical study (*n* = 283) found no significant improvement in suicidality (or affective symptoms) with the use of adjunctive low-level lithium (mean 0.47mmol/L) in symptomatic bipolar patients compared to usual care [21]. Of note, the sample had high rates of comorbidities, ongoing episodes from baseline and those randomised to lithium (as opposed to ‘optimised usual care’) may have had a more severe mania burden. Distinct from low dosing, is *trace* dose lithium (< 5 mg ionic lithium), which has its own body of ecological evidence suggesting its benefits on the effects of suicidality [22] though clearly, the quality of evidence behind lithium’s benefits on suicidality from such studies is limited.

### Mania

A few studies have reported notable benefits to mania, one appearing comparable to standard dose lithium (both achieving a response rate of 21%) [23] and another reporting benefits compared to placebo [24], although it must be highlighted that in many cases, higher doses will be required at least for a brief period.

Together, the evidence for low dose lithium across neuropsychiatric health has recently been systematically reviewed [25]. Being that the most consistent finding from this related to low-dose lithium benefits to cognitive functioning, it is worth mentioning here other indications for low dose lithium. Even at low doses lithium appears to have biological effects that may be neuroprotective [26] and it is worth mentioning other putative indications for low doses including relating to dementia [27], suicide [22] and various physical outcomes [28].

Low-dose lithium can be combined with an additional mood stabiliser at just above its minimum therapeutic level to maintain effectiveness with increased tolerability of both medications [29].

Box 2 summarises one real-world clinical case using lower dose lithium with apparent benefits.

**Table:** 

Case 2:
*eGFR gradually reducing (< 60) while lithium levels were* *≥* *0.6mmol/L. Lithium was reduced to levels 0.4-0.4.5mmol/L; within this range*,* eGFR fluctuated somewhat but did not progress*,* staying ~ 75–80. Patient required concomitant valproate at lower doses for affective stability. The patient had previously had frequent hospitalisations*,* an alcohol use disorder (precipitated by affective switch - mania or depression)*,* which had exacerbated depressive (e.g.*,* suicidality) and manic (e.g.*,* reckless behaviour) episode severity. Lithium theorised to actively treat the alcohol use disorder in addition to affect; reduction in lithium dose was reported to be a specific motivating factor for lifestyle changes (abstinence from alcohol*,* smoking*,* plus diet) in order to achieve persistent recovery*,* which has been successful to date.*

## Start Low and Go Slow

Reverting back to lithium’s primary indication of mood stabilisation, it is clear that higher doses are more effective (but more harmful) and vice versa. However, we also are aware that for some individuals with affective illness, a low dose is clinically effective. We do not possess strong evidence regarding for whom that is the case. In this case, we might consider the potential value of a lithium initiation strategy that may yield clinical benefits without adverse effects for some: *start low and go slow.*

There are no large-scale, head-to-head clinical trials that directly compare the ‘*start low and go slow’* titration strategy to the more traditional or standard titration techniques in a systematic, controlled manner.

We are aware however, of some centres that have implemented this. In a primary care led Collaborative Care Program in Oregon, some patients with affective disorders (usually presumed bipolar type II or bipolar spectrum) are initiated on 150 mg lithium and titrated in 150 mg increments every 4–7 days until either a clinical response or side effects are observed (in the latter case, the dose is then lowered by one step), usually up to a maximum 450–600 mg. In a naturalistic sample of lithium-treated patients from this clinic, the mean lithium serum level was 0.43mmol/L and 84% were taking *≤* 600 mg/day. Symptoms of depression and anxiety were reduced in lithium (and a group taking lamotrigine) compared to those undergoing other treatments; clinically important differences were reported at a rate of 49% (depression) and 44% (anxiety) after three months [30]. Although these benefits do not appear striking, it has also been reported that the regimen has been highly acceptable and widely adopted within the aforementioned centre [30]. Furthermore, it appears to substantially minimise side effect burdens [31].

## Dose Reduction during Periods of Stability

Dose modification clearly is not suitable for all patients and must be personalised, based on a patient’s unique profile including the observed natural history of the condition, the degree of risk when unwell, their preferences, physical health status and experience of side effects. Indeed, variable dosing based on current wellness and risk of relapse is another strategy which is not empirically validated. However, we know that:

- Many people with bipolar disorders are able to sustain long periods of time without affective symptoms, particularly while taking lithium [32].
- Reduced dose and/or frequency of lithium is associated with reduced adverse renal effects [33, 34].
- Gradual down-titration appears to substantially reduce the time to symptom recurrence; even when ultimately discontinued, one study has reported a time to relapse of 2.5 months when lithium was rapidly discontinued, compared with 14 months when gradually discontinued [35].
- Empowerment in managing one’s own treatment is evidenced to improve acceptability and adherence [36].
- There are other interventions, which could be employed concomitantly with lithium, that decrease relapse through enhanced recognition of episode risk e.g., early warning signs training [37].

Thus, for some patients, lithium dose could be maintained at a lower level when stably euthymic and increased when at risk of relapse or upon the emergence of subsyndromal symptoms. For these patients, dose modifications should be clinician-led and with agreement between both the patient and clinician as to the logistics and level of autonomy in dose management.

It is anticipated that this approach may not be suitable for people who experience high rates of relapse or ongoing inter-episode symptoms. These individuals may require maintenance of a lithium level within 0.6-0.6.8mmol/l (compared to 0.4–0.5 mmol/l). It is perhaps those (often with bipolar type I) who do not experience more than trivial symptoms when between episodes who could benefit from lithium levels between 0.4-0.4.5mmol/L when well.

A proposition is that, prior to any dose reduction, and duration a period of euthymia, a patient can be trained to recognise early warning signs of an impending relapse. Some common early warning signs include an increase in severity and/or duration of any usual inter-episode symptoms, however many individuals will have specific personal warning signs (e.g., an increase in substance use, impulsive spending). If agreed by clinician and patient following early warning signs training, a gradual down titration could be implemented (e.g., over four weeks) to the reduced level. Subsequently, upon the emergence of warning signs or risk of relapse recognition, an agreed strategy can then be implemented. This agreed strategy could include increased lithium dose resulting in a blood level 0.6–0.8 or even above this, maintained until remission or a number of weeks sustained remission. It may be that for mania, this is combined with a low dose of an antimanic such as 2.5 mg olanzapine, or a sedative. Here, it is worth noting that although lithium is widely considered to work more slowly than other mood stabilisation treatments, but it is also evidenced to have rapid efficacy against both mania and depression [38].

To summarise, evidence could suggest that – for some individuals – lithium treatment could be maintained with clinical stability at a lower dose until a time where an increased dose is warranted (e.g., risk of episode, subsyndromal symptoms) at which point the dose is increased until needed, and then gradually reduced once more. If so, this could maintain lithium’s overall mood stabilisation effect while increasing acceptability reducing adverse effects.

Box 3 summarises one real-world clinical case using variable lithium dosing with apparent benefits.

**Table:** 

Case 3:
Patient experienced prolonged and intolerable affective blunting with levels > 0.6 mmol/L. When reduced to 0.4–0.5 mmol/L</i>,<i>affective blunting was minimal. Group psychoeducation and training in early warning signs (of a potential relapse) allowed the lower level to be maintained except when early warning signs detected at which time lithium dose is increased. This is considered to have been successful – the patient had 3 hospital admissions before lithium initiation and 0 since. On one occasion</i>,<i>quetiapine added to lithium during relapse.

## New Techniques to Enhance Lithium Monitoring

Individuals with lithium levels below the therapeutic range have a greater risk of relapse, while those with levels above this range face an elevated risk of side effects and lithium toxicity [39]. In addition to dose, factors that alter electrolyte levels, such as other agents which alter lithium excretion, dehydration, and infections can contribute to lithium intoxication [40]. Therefore, frequent monitoring of serum levels is vital throughout maintenance treatment with lithium. Notwithstanding this importance, in many parts of the world, it appears that only 30–50% of lithium-treated patients undergo adequate monitoring [5, 41, 42]. Several of the reasons for this may be logistical e.g., challenges communicating results between clinical teams [5].

Emerging technologies for lithium monitoring show significant potential to enhance the safety and efficacy of lithium therapy in psychiatric disorders. We highlight that this is a future potential as opposed to a current recommendation.

Saliva testing, for instance, offers a non-invasive and patient-friendly alternative to traditional blood draws. This method, which has been tested in clinical studies, incorporates clinical and environmental covariates to improve predictive accuracy; however, factors such as salivary flow rate and patient hydration may influence the reliability of lithium measurements [43]. Textile-based sensors, integrated into wearable devices, have been tested in pre-clinical proof-of-concept studies. These sensors enable continuous, non-invasive monitoring of lithium levels, offering real-time data collection, but they face challenges related to long-term stability, calibration, and reproducibility in real-world settings [44]. Reverse iontophoresis, also in the pre-clinical phase, shows promise for transdermal lithium monitoring by enabling pharmacokinetic profiling in a non-invasive manner. However, this technique requires further optimization to ensure consistent lithium extraction across different skin types and conditions [45]. Electrochemical sensing systems, tested in pre-clinical studies, represent another promising approach for non-invasive lithium monitoring. These systems are integrated into wearables, facilitating real-time adjustments to lithium therapy, but still require refinement to achieve robustness and reliability for clinical use [46]. Lastly, hydrogel-forming microneedle arrays, currently in the pre-clinical phase, offer a minimally invasive method for lithium detection, combining ease of use with reliable data collection. However, their long-term usability, patient compliance, and potential for skin reactions need further investigation before they can be applied in clinical practice [47]. While these emerging techniques offer innovative solutions for lithium monitoring, they require further clinical validation to address these limitations and to ensure their integration into routine clinical practice.

Four factors to be considered in the optimisation of these pathways include:

- Invasiveness: Saliva testing and textile-based sensors are completely non-invasive, while microneedles and reverse iontophoresis are minimally invasive or non-invasive but require some form of interaction with the skin.
- Type of bodily fluid or method: Saliva testing uses saliva, microneedles sample interstitial fluid, and iontophoresis and electrochemical systems usually target sweat or other skin-extracted fluids. Textile-based systems rely on real-time monitoring through embedded sensors.
- Stage of development: Saliva testing is already in clinical use, while the other four methods are in various pre-clinical stages, focusing on proof-of-concept studies and early feasibility testing.
- Monitoring capability: Textile-based sensors, reverse iontophoresis, and electrochemical sensing systems have the potential for continuous monitoring, while saliva testing and microneedles are more suited for periodic measurements.

In addition, we highlight that advances in monitoring techniques need to be considered in terms of maintaining or improving reporting frameworks and procedures to ensure that results are delivered to the right person in a timely manner such that optimised action can be taken.

## Closing Points

The authors highlight that the evidence synthesised in this piece was not conducted as a systematic review. We also emphasise that the article primarily focuses on bipolar disorders as lithium’s primary indication; although some material applies across populations (e.g., unipolar depressive or neurodegenerative conditions), the content here is not fully generalisable.

As well as the above suggested strategies, we must bear in mind that there are many more approaches to be considered. The first, is to ensure clinical practice meets clinical demands: The importance of proper medical screening (calcium, thyroid, renal, cardiac), proper monitoring, management of polypharmacy and contraindications should not be underestimated. Beyond this, we should consider the benefits of specifics such as: increased monitoring in certain cases or at certain times [48]; alternative formulations [49]; once (versus twice) daily dosing [50] and patient education [51]. Precision psychiatry will likely assist with predicting candidates for excellent or poor lithium response a priori and we know already of factors associated with response to lithium (e.g., family history of lithium response) [52].

## Data Availability

No datasets were generated or analysed during the current study.

## References

[1] Malhi GS, Bauer M. Lithium first: not merely first line. Bipolar Disord. Feb. 2023;25(1):7–8. 10.1111/bdi.13299. 10.1111/bdi.13299 36808784

[2] Strawbridge R, Young AH. Lithium: how low can you go? Int J Bipolar Disord. 2024;12(1):4. 38289425 10.1186/s40345-024-00325-y PMC10828288

[3] Meyer JM, Stahl SM, editors. ‘The Lithium Handbook: Stahl’s Handbooks’, in *The Lithium Handbook: Stahl’s Handbooks*, in Stahl’s Essential Psychopharmacology Handbooks., Cambridge: Cambridge University Press, 2023, pp. iii–iii. Accessed: Nov. 04, 2024. [Online]. Available: https://www.cambridge.org/core/books/lithium-handbook/lithium-handbook/85FF7B5B02701AEBE02E2BAEC536E541

[4] Strawbridge R, Young AH. Lithium: balancing mental and renal health. Lancet Psychiat. 2022;9(10) 760-761 10.1016/S2215-0366(22)00308-X 10.1016/S2215-0366(22)00308-X 36108667

[5] Collins N, Barnes TR, Shingleton-Smith A, Gerrett D, Paton C. Standards of lithium monitoring in mental health trusts in the UK. BMC Psychiat. 2010;10(1):80. 10.1186/1471-244X-10-80. 10.1186/1471-244X-10-80 PMC2958995 20939864

[6] Öhlund L, et al. Reasons for lithium discontinuation in men and women with bipolar disorder: a retrospective cohort study. BMC Psychiatr. 2018;18(1):37. 10.1186/s12888-018-1622-1. 10.1186/s12888-018-1622-1 PMC5804058 29415689

[7] Canning JE, Burton S, Hall B. Lithium and valproate-induced tremors. Mental Health Clinician. 2012;1(7):174–6.

[8] Gelenberg AJ, Jefferson JW. ‘Lithium tremor’, J Clin Psychiatr. 1995;56(7)283–287. 7615481

[9] Batlle DC, von Riotte AB, Gaviria M, Grupp M. Amelioration of polyuria by amiloride in patients receiving long-term lithium therapy. N Engl J Med. 1985; 312(7)408–414. 10.1056/NEJM198502143120705 10.1056/NEJM198502143120705 3969096

[10] Strawbridge R, Young A, Prabhu A, Abbott M, Meyer J. ‘ENaC inhibitors for the management of lithium related polyuria: a systematic review’. J Affect Disord. 2025 May 29:119542. 10.1016/j.jad.2025.119542 10.1016/j.jad.2025.119542 40449743

[11] Vandermeulen L, Van Melkebeke L, Sienaert P. Lithium-associated hypercalcemia and hyperparathyroidism: A systematic review and meta-analysis. World J Biol Psychiat. 2024;25(8) 417–429. 10.1080/15622975.2024.2393373 10.1080/15622975.2024.2393373 39192574

[12] Pattan V, Singh B, Abdelmoneim SS, Gopinath C, Sundaresh V. Lithium-Induced Hyperparathyroidism: An Ill-defined Territory. Psychopharmacol Bull. 2021;5(3):65. 10.64719/pb.4410 PMC8374931 34421145

[13] Cowdry RW, Wehr TA, Zis AP, Goodwin FK. Thyroid Abnormalities Associated With Rapid-Cycling Bipolar Illness. Arch Gen Psychiat. 1983;40(4):414–20. 10.1001/archpsyc.1983.01790040068010. 6404231 10.1001/archpsyc.1983.01790040068010

[14] Lazarus JH. The effects of lithium therapy on thyroid and thyrotropin-releasing hormone. Thyroid Off J Am Thyroid Assoc. 1998;8(10):909–13. 10.1089/thy.1998.8.909. 10.1089/thy.1998.8.909 9827658

[15] Steardo L, et al. Clinical severity and calcium metabolism in patients with bipolar disorder. Brain Sci. 2020;10(7):417. 10.3390/brainsci10070417. 10.3390/brainsci10070417 PMC7408522 32630307

[16] Schou M, Juel-Nielsen N, Stromgren E, Voldby H. The treatment of manic psychoses by the administration of lithium salts. J Neurol Neurosurg Psychiat. 1954; 17(4)250–260. 10.1136/jnnp.17.4.250 10.1136/jnnp.17.4.250 PMC503195 13212414

[17] Mendelsohn A. The time psychiatry forgot about notation: the 12-hour lithium level. Anxious Phys. Accessed: Jan. 17, 2025. [Online]. Available: https://theanxiousphysicist.com/the-time-psychiatry-forgot-about-notation-the-12-hour-lithium-level

[18] Ercis M, Ozerdem A, Singh B. When and how to use lithium augmentation for treating major depressive disorder. J. Clin Psychiatry. 2023; 84(2)23ac14813. 10.4088/JCP.23ac14813 10.4088/JCP.23ac14813 36883886

[19] Scott F, et al. Systematic review and meta-analysis of augmentation and combination treatments for early-stage treatment-resistant depression. J Psychopharmacol (Oxf). 2022;02698811221104058. 10.1177/02698811221104058. 10.1177/02698811221104058 PMC10076341 35861202

[20] Bauer M, Dopfmer S. Lithium augmentation in treatment-resistant depression: meta-analysis of placebo-controlled studies. J Clin Psychopharmacol. 1999; 19(5)427–434. 10.1097/00004714-199910000-00006 10.1097/00004714-199910000-00006 10505584

[21] Nierenberg AA et al. Lithium treatment -- moderate dose use study (LiTMUS) for bipolar disorder: rationale and design. Clin Trials. 6(6)637–648. 10.1177/1740774509347399 19933719

[22] Memon A, et al. Association between naturally occurring lithium in drinking water and suicide rates: systematic review and meta-analysis of ecological studies. Br J Psychiatry. 2020;217(6):667–78. 32716281 10.1192/bjp.2020.128

[23] Stokes PE, Kocsis JH, Arcuni OJ. Relationship of lithium chloride dose to treatment response in acute mania. Arch Gen Psychiatry. 1976;33(9):1080–4. 17642108 10.1001/archpsyc.1976.01770090070006

[24] Nagel K, Adler LE, Bell J, Nagamoto HT, Freedman R. Lithium carbonate and mood disorder in recently detoxified alcoholics: A double-blind, placebo-controlled pilot study. Alcohol Clin Exp Res. 1991;15(6)982–90. 10.1111/j.1530-0277.1991.tb05198.x 1789394

[25] Strawbridge R, et al. Identifying the neuropsychiatric health effects of low-dose lithium interventions: A systematic review. Neurosci Biobehav Rev. 2023;144:104–975. 10.1016/j.neubiorev.2022.104975. 10.1016/j.neubiorev.2022.104975 36436738

[26] Neal MA, Strawbridge R, Wing VC, Cousins DA, Thelwall PE. Human brain 7Li-MRI following low-dose lithium dietary supplementation in healthy participants. J Affect Disord. 2024, Accessed: Nov. 04, 2024. [Online]. Available: https://www.sciencedirect.com/science/article/pii/S0165032724008723 10.1016/j.jad.2024.05.128 38810780

[27] Kessing LV, et al. Association of lithium in drinking water with the incidence of dementia. JAMA Psychiatry. 2017;74(10):1005. 10.1001/jamapsychiatry.2017.2362. 10.1001/jamapsychiatry.2017.2362 PMC5710473 28832877

[28] Hamstra SI, et al. Beyond its psychiatric use: the benefits of Low-dose lithium supplementation. Curr Neuropharmacol. 2023;21(4):891. 10.2174/1570159X20666220302151224. 10.2174/1570159X20666220302151224 PMC10227915 35236261

[29] ‘Lithium plus valproate combination therapy versus monotherapy for relapse prevention in bipolar I disorder (BALANCE): a randomised open-label trial’, The Lancet, vol. 375, no. 9712, pp. 385–395. Jan. 2010, 10.1016/S0140-6736(09)61828-6 10.1016/S0140-6736(09)61828-6 20092882

[30] Phelps JR, Pipitone OR, Squires K, Bale JD. Lamotrigine and lithium in primary care psychiatric consultation: adoption and adverse effects. Fam Pract. 2021; 38(4) 381–386. 10.1093/fampra/cmaa131 10.1093/fampra/cmaa131 33367908

[31] Phelps J, Coskey OP. Low and very low lithium levels: thyroid effects are small but still require monitoring. Bipolar Disord. 2024;26(2):129–35. 10.1111/bdi.13377. 37704933 10.1111/bdi.13377

[32] Ulrichsen A, Hampsey E, Taylor RH, Gadelrab R, Strawbridge R, Young AH. Comparing measurements of lithium treatment efficacy in people with bipolar disorder: systematic review and meta-analysis. BJPsych Open 2023;9(3):e98. 10.1192/bjo.2023.64. 10.1192/bjo.2024.807 PMC11698155 39534908

[33] Singh LK, Nizamie SH, Akhtar S, Praharaj SK. Improving tolerability of lithium with a once-daily dosing schedule. Am J Ther. 2011;18(4):288–91. 10.1097/MJT.0b013e3181d070c3. 10.1097/MJT.0b013e3181d070c3 20592663

[34] Aprahamian I, et al. Long-term, low-dose lithium treatment does not impair renal function in the elderly: a 2-year randomized, placebo-controlled trial followed by single-blind extension. J Clin Psychiatry. 2014;75(7):e672–678. 10.4088/JCP.13m08741. 10.4088/JCP.13m08741 25093483

[35] Baldessarini RJ, Tondo L, Floris G, Rudas N. Reduced morbidity after gradual discontinuation of lithium treatment for bipolar I and II disorders: a replication study. Am J Psychiatry. 1997; 154(4) 551–553. 10.1176/ajp.154.4.551 10.1176/ajp.154.4.551 9090345

[36] Náfrádi L, Nakamoto K, Schulz PJ. Is patient empowerment the key to promote adherence? A systematic review of the relationship between self-efficacy, health locus of control and medication adherence. PLoS One. 2017;12(10):e0186458. 10.1371/journal.pone.0186458. 10.1371/journal.pone.0186458 PMC5645121 29040335

[37] Morriss RK, Faizal MA, Jones AP, Williamson PR, Bolton C, McCarthy JP. Interventions for helping people recognise early signs of recurrence in bipolar disorder. Cochrane Database Syst. Rev. 2007; (1)CD004854. 10.1002/14651858.CD004854.pub2 10.1002/14651858.CD004854.pub2 PMC6544804 17253526

[38] Keck PE et al. A pilot study of rapid lithium administration in the treatment of acute mania. Bipolar Disord.2001; 3(2)68–72. 10.1034/j.1399-5618.2001.030204.x 10.1034/j.1399-5618.2001.030204.x 11333065

[39] Severus WE, Kleindienst N, Seemüller F, Frangou S, Möller HJ, Greil W. What is the optimal serum lithium level in the long-term treatment of bipolar disorder–a review?’. Bipolar Disord. 2008; 10(2) 231–237. 10.1111/j.1399-5618.2007.00475.x 10.1111/j.1399-5618.2007.00475.x 18271901

[40] Gitlin M. Lithium side effects and toxicity: prevalence and management strategies. Int J Bipolar Disord. 2016;4(1):27. 10.1186/s40345-016-0068-y. 10.1186/s40345-016-0068-y PMC5164879 27900734

[41] Nikolova VL, Pattanaseri K, Hidalgo-Mazzei D, Taylor D, Young AH. Is lithium monitoring NICE? Lithium monitoring in a UK secondary care setting. J Psychopharmacol Oxf Engl. 2018;32(4) 408–415. 10.1177/0269881118760663 10.1177/0269881118760663 29552933

[42] Paton C, Adroer R, Barnes TRE. Monitoring lithium therapy: the impact of a quality improvement programme in the UK. Bipolar Disord. 2013; 15(8)865–875. 10.1111/bdi.12128 10.1111/bdi.12128 24119180

[43] Parkin GM, et al. Saliva testing as a means to monitor therapeutic lithium levels in patients with psychiatric disorders: identification of clinical and environmental covariates, and their incorporation into a prediction model. Bipolar Disord. 2021;23(7):679. 10.1111/bdi.13128. 10.1111/bdi.13128 PMC9291088 34536974

[44] Sweilam MN, et al. Textile-based non-invasive lithium drug monitoring: A proof-of-concept study for wearable sensing. Biosens Bioelectron. 2020;150:111-897. 10.1016/j.bios.2019.111897. 10.1016/j.bios.2019.111897 31786018

[45] Leboulanger B, Fathi M, Guy RH, Delgado-Charro MB. Reverse iontophoresis as a noninvasive tool for lithium monitoring and Pharmacokinetic profiling. Pharm Res 2004;21:1214–22. 10.1023/B:PHAM.0000033008.64915.c8. 10.1023/b:pham.0000033008.64915.c8 15290862

[46] Criscuolo F, Cantù F, Taurino I, Carrara S, De Micheli G. A wearable electrochemical sensing system for non-invasive monitoring of lithium drug in bipolar disorder. IEEE Sens J. 2021; 21(8) 9649–9656. 10.1109/JSEN.2020.3009538

[47] Eltayib E, et al. Hydrogel-forming microneedle arrays: potential for use in minimally-invasive lithium monitoring. Eur J Pharm Biopharm. 2016;102:123–31. 10.1016/j.ejpb.2016.03.009. 26969262 10.1016/j.ejpb.2016.03.009

[48] Heald AH et al. Can we check serum lithium levels less often without compromising patient safety?’, BJPsych Open. 2021; 8(1)e18. 10.1192/bjo.2021.1027 10.1192/bjo.2021.1027 PMC8715256 34915951

[49] Taylor DM, Barnes TRE, Young AH. The Maudsley Prescribing Guidelines in Psychiatry. Wiley; 2021.

[50] Plenge P, Mellerup ET. Lithium and the kidney: Is one daily dose better than two? Compr Psychiat. 1986;27(4)336–342. 10.1016/0010-440X(86)90009-X 10.1016/0010-440x(86)90009-x 3731768

[51] Zolezzi M, Eltorki YH, Almaamoon M, Fathy M, Omar NE. Outcomes of patient education practices to optimize the safe use of lithium: A literature review. Ment Health Clin. 2018;8(1):41–8. 10.9740/mhc.2018.01.041. 29955544 10.9740/mhc.2018.01.041 PMC6007520

[52] Scott J, et al. Prospective cohort study of early biosignatures of response to lithium in bipolar-I-disorders: overview of the H2020-funded R-LiNK initiative. Int J Bipolar Disord. 2019;7(1):20. 10.1186/s40345-019-0156-x. 31552554 10.1186/s40345-019-0156-x PMC6760458

